# A Simple and Fast Spline Filtering Algorithm for Surface Metrology

**DOI:** 10.6028/jres.120.010

**Published:** 2015-07-15

**Authors:** Hao Zhang, Daniel Ott, John Song, Mingsi Tong, Wei Chu

**Affiliations:** 1 College of Mechanical and Electronic Engineering, Nanjing Forestry University, Nanjing, 210037, China; 2 National Institute of Standards and Technology, Gaithersburg, MD 20899 USA; 3 School of Mechatronics Engineering, Harbin Institute of Technology, Harbin, 150001, China

**Keywords:** discrete cosine transform, discrete Fourier transform, spline filter, surface metrology

## Abstract

Spline filters and their corresponding robust filters are commonly used filters recommended in ISO (the International Organization for Standardization) standards for surface evaluation. Generally, these linear and non-linear spline filters, composed of symmetric, positive-definite matrices, are solved in an iterative fashion based on a Cholesky decomposition. They have been demonstrated to be relatively efficient, but complicated and inconvenient to implement. A new spline-filter algorithm is proposed by means of the discrete cosine transform or the discrete Fourier transform. The algorithm is conceptually simple and very convenient to implement.

## 1. Introduction

A typical engineering surface consists of a range of spatial frequencies. The high frequency or short wavelength components are referred to as roughness, the medium frequencies as waviness and low frequency components as form [1, 2]. Filtration is a critical process used to isolate form, waviness, and surface roughness from each other as well as from finer, higher frequency content such as instrument noise [3]. At present, the Gaussian filter, the spline filter and their corresponding robust filters are recommended as ISO standard profile filters [4–7]. Compared with the widely used Gaussian filter, the spline filters have advantages such as end preserving, efficient calculation and good form following.

Generally, the spline filter is implemented using an iterative procedure based on a Cholesky matrix decomposition [2, 8]. It is a widely used and relatively efficient method for solving the spline filter. In this paper, a new approach for computing the spline filter is proposed that relies on the discrete cosine transform (DCT) or the discrete Fourier transform (DFT) rather than matrix decomposition. This new algorithm is conceptually more simple, efficient, and, in practice, more convenient to implement.

## 2. The Spline Filters

### 2.1 Linear Spline Filter

In ISO/TS 16610-22 [5], the standard spline filter is given as

$$(I+\beta \alpha^{2}P+(1-\beta )Q)w=z$$ (1)

where ***I*** is the identity matrix, ***Z*** is the vector of sampled data values, ***W*** is the vector of output data values, and ***P*** and ***Q*** are the coefficient matrices The spline filter of Eq. (1) is obtained as the solution matrix of the classic variational method in Eq. (2) [8–9].

$$\min\left({\left\|z-w\right\|}^{2}+\beta \alpha^{2}{\left\|D_{1}z\right\|}^{2}+\left(1-\beta \right){\left\|D_{2}z\right\|}^{2}\right)$$ (2)

where ‖·‖ denotes the Euclidean norm (L_2_ norm), and *D*_1_ and *D*_2_ stand for 1^st^ and 2^nd^ differential operators. Generally, Eq. (2) is described as a linear combination of a least squares approach and a penalty component consisting of two differential terms. More specifically, the least squares term aims to guarantee a close match between the result ***W*** and the profile ***Z*** [10], and the penalty component helps to ensure appropriate smoothness of the filtered result. The scalars *α* and *β* are used to determine the balance between closeness to the original data and amount of smoothness, where $\alpha ={\left(2\sin\left(\pi /\lambda_{c}\right)\right)}^{-1}$ (*λ_c_* is the cut-off wavelength) and 0 ≤ *β* ≤1 [5]. Since all terms of Eq. (2) are differentiable, the matrix equation of Eq. (1) is obtained directly by simple mathematical derivation. Here it should be noted that the boundary conditions, which are dictated by *P* and *Q*, vary depending on the type of surface profile, which may be classified as periodic or non-periodic (see Eqs. (3) and (4)).

$$P=\{\begin{array}{l} \left(\begin{array}{ccccc} 1 & -1 & & & \\ -1 & 2 & -1 & & \\ & \ddots & \ddots & \ddots & \\ & & -1 & 2 & -1\\ & & & -1 & 1 \end{array}\right)\text{non-periodic}\\ \left(\begin{array}{ccccc} 2 & -1 & & & -1\\ -1 & 2 & -1 & & \\ & \ddots & \ddots & \ddots & \\ & & -1 & 2 & -1\\ -1 & & & -1 & 2 \end{array}\right)\text{periodic} \end{array}$$ (3)

$$Q=\{\begin{array}{l} \left(\begin{array}{ccccccc} 1 & -2 & 1 & & & & \\ -2 & 5 & -4 & 1 & & & \\ 1 & -4 & 6 & -4 & 1 & & \\ & \ddots & \ddots & \ddots & \ddots & \ddots & \\ & & 1 & -4 & 6 & -4 & 1\\ & & & 1 & -4 & 5 & -2\\ & & & & 1 & -2 & 1 \end{array}\right)\text{non-periodic}\\ \left(\begin{array}{ccccccc} 6 & -4 & 1 & & & 1 & -4\\ -4 & 6 & -4 & 1 & & & 1\\ 1 & -4 & 6 & -4 & 1 & & \\ & \ddots & \ddots & \ddots & \ddots & \ddots & \\ & & 1 & -4 & 6 & -4 & 1\\ 1 & & & 1 & -4 & 6 & -4\\ -4 & 1 & & & 1 & -4 & 6 \end{array}\right)\text{periodic} \end{array}$$ (4)

### 2.2 Robust Spline Filter

It is well known that the linear filter is vulnerable to outliers [11]. Although there is no agreement on a universal and formal definition of an outlier, it is usually regarded as the observations that lie abnormally far from others, such as scratches and deep valleys on a surface. The roughness profiles or surfaces will be distorted when the linear filtering algorithm is applied to this original sampling data containing outliers. The resulting filtered data will contain excessively swollen or sunk areas due to being “pulled down or up” in the vicinity of abnormal points. This phenomenon will certainly cause a serious error when performing the evaluation or analysis of a practical surface.

In order to solve this problem, the concept of the robustness of a filtering algorithm is introduced. In a robust filter, the effect of outliers on surrounding data is mitigated. Both L_1_-norm and L_2_-norm based robust spline filters have been demonstrated [7, 12]. A robust spline filter based on the L_2_-norm [12] is obtained by the direct expansion of Eq. (1), that is:

$$(\delta +\beta \alpha^{2}P+\left(1-\beta \right)\alpha^{4}Q)w=\delta z$$ (5)

where ***P*** and ***Q*** are the same coefficient matrices as in Eqs. (3) and (4). ***δ*** contains the additional weights corresponding to each data point of *z. **δ*** is updated with a specified weighting function by using current residuals from iteration to iteration, until the residuals remain unchanged [13]. Several weighting functions are available such as Huber, Hampel, and Andrews. Indeed, the most widely used weighting function in surface metrology is the Tukey function [6].

## 3. Simplified Algorithm

### 3.1 The Spline Filter for Non-Periodic Profiles

As previously mentioned, the standard spline filter is endowed with different solution matrices based on the periodicity of the profile and how this is handled at boundaries. Let us first focus on the matrix ***Q*** under the non-periodic condition, which is also called the natural boundary, that is, when the 2^nd^ differential equation equals zero. If we instead redefine the boundary condition as the 1^st^ differential equation equaling zero, the original matrix ***Q*** can be simplified and rewritten in the following form

$$Q=\left(\begin{array}{ccccccc} 2 & -3 & 1 & & & & \\ -3 & 6 & -4 & 1 & & & \\ 1 & -4 & 6 & -4 & 1 & & \\ & \ddots & \ddots & \ddots & \ddots & \ddots & \\ & & 1 & -4 & 6 & -4 & 1\\ & & & 1 & -4 & 6 & -3\\ & & & & 1 & -3 & 2 \end{array}\right).$$ (6)

It can be easily verified that ***Q*** = ***D^T^D***,

$$D=\left(\begin{array}{ccccc} -1 & 1 & & & \\ 1 & -2 & 1 & & \\ & \ddots & \ddots & \ddots & \\ & & 1 & -2 & 1\\ & & & 1 & -1 \end{array}\right)$$ (7)

where ***D*** is a tridiagonal matrix and ***D^T^*** represents the transpose of ***D***

Furthermore, an eigen decomposition of ***D*** yields ***D*** = ***U******⋀******U**^T^* [13]. ***⋀*** is a diagonal matrix containing the eigenvalues of ***D*** [14]

$$\Lambda =\text{diag}(\lambda_{1},\ldots ,\lambda_{n})\;with\;\lambda_{i}=-2+2\cos((i-1)\pi /n)$$

where *n* is the number of sampled points.

Since ***U*** is a unitary matrix, Eq. (1) can be written as

$$w={\left(I-\beta \alpha^{2}D+\left(1-\beta \right)D^{T}D\right)}^{-1}z=U{\left(I-\beta \alpha^{2}\Lambda +\left(1-\beta \right)\Lambda^{2}\right)}^{-1}U^{T}z$$ (8)

According to Reference [15], ***U**^T^* is a type-2 DCT matrix and ***U*** is an inverse DCT matrix. Let us define $\Gamma =\text{diag}\left(\Gamma_{1},\ldots ,\Gamma_{n}\right)$ with $\Gamma_{i}={\left[1-\beta \alpha^{2}\lambda_{i}+\left(1-\beta \right)\lambda_{i}^{2}\right]}^{-1}$, Eq. (8) can be rewritten in the form

$$w={\text{DCT}}^{-1}\left(\Gamma \text{DCT}\left(z\right)\right)$$ (9)

where DCT^−1^ represents the inverse discrete cosine transform.

In fact, DCT is a Fourier-related transform similar to DFT, a technique for mapping the “time domain” to the “frequency domain.” Both DCT and DFT express a signal in terms of a sum of sinusoids with different frequencies and amplitudes. The distinction between DFT and DCT is that, DFT uses both cosine and sine functions, while DCT uses only cosine functions [16]. Boundary conditions have a direct impact on the form of discrete transfer functions such that different combinations of the discrete boundary conditions give rise to eight different types of DCT. Of these eight DCT representations, the most commonly used transform is type-2 [16].

### 3.2 The Spline Filter for Periodic Profiles

For the case of a periodic profile, the boundary conditions will require that matrix ***Q*** refer to the classic assumption of circular data defined as *Z_i_* = *Z*_*i*_+*n*, that is

$$Q=\left(\begin{array}{ccccccc} 6 & -4 & 1 & & & 1 & -4\\ -4 & 6 & -4 & 1 & & & 1\\ 1 & -4 & 6 & -4 & 1 & & \\ & \ddots & \ddots & \ddots & \ddots & \ddots & \\ & & 1 & -4 & 6 & -4 & 1\\ 1 & & & 1 & -4 & 6 & -4\\ -4 & 1 & & & 1 & -4 & 6 \end{array}\right).$$ (10)

One can obtain the same description ***Q*** = ***D^T^D***, where ***D*** is also a circulant matrix,

$$D=\left(\begin{array}{ccccc} -2 & 1 & & & 1\\ 1 & -2 & 1 & & \\ & \ddots & \ddots & \ddots & \\ & & 1 & -2 & 1\\ 1 & & & 1 & -2 \end{array}\right).$$ (11)

For this case, a DCT is not able to fulfill the spline filter equation because the boundary definition for any type of DCT does not comply with a periodic boundary of *Z_i_* = *Z_i_*+*n* However, it is interesting that this boundary condition is exactly the same as the boundary assumption of the DFT. According to signal processing theory, a discrete spectrum necessarily comes from a periodic signal in the time domain. Hence, it is rational to apply the fast Fourier transform (FFT) to the computation of the spline filter given a periodic boundary. As expected, following the related derivation shown in Section 3.1, we obtain

$$w={\text{FFT}}^{-1}\left(\Gamma \text{FFT}\left(z\right)\right)$$ (12)

where FFT^−1^ stands for the inverse fast Fourier transform.

## 4. Robust Simplified Algorithm

As previously mentioned, the sensitivity of the linear filter to outliers may result in the mean line deviating severely from the actual distribution. The occurrence of dropouts, e.g., resulting from an instrument’s inability to measure certain data features, is frequent in practice [13]. Fortunately, the weighting function is demonstrated to be an effective method for handling such dropouts. This function serves the purpose of giving outliers a low weight and dropouts a weight of zero, while allocating a relatively high weight to high-quality data.

For convenience, Eq. (5) can be rewritten as

$$(I+\beta \alpha^{2}P+(1-\beta )\alpha^{4}Q)w=(I-\delta )w+\delta z.$$ (13)

This implicit formula can be solved using an iterative procedure

$$w^{k+1}={\left(I+\beta \alpha^{2}P+(1-\beta )\alpha^{4}Q\right)}^{-1}(\delta (z-w^{k})+w^{k})$$ (14)

where ***W**^k^*+1 refers to ***W**^k^* at the *k*th iteration step. Furthermore, referring to Eqs. (9) and (12) for the appropriate boundary conditions, we can easily obtain the new equations

$$w^{k+1}={\text{DCT}}^{-1}(\Gamma \text{DCT}(\delta (z-w^{k})+w^{k}))\;\text{for the non-periodic case},$$ (15)

or

$$w^{k+1}={\text{FFT}}^{-1}(\Gamma \text{FFT}(\delta (z-w^{k})+w^{k}))\;\text{for the periodic case}.$$ (16)

The robustness to outliers is realized by iterating the weighted function of Eq. (15) or (16) and updating the weights using robust estimators for the current residuals (***z*** − ***W**^k^*) It should be noted that during one iteration, the DCT and FFT only require *n*log(*n*) operations for a profile *n* data points, whereas Eq. (1) requires a Cholesky factorization with computational complexity of *O*(*n*^3^) [13].

## 5. Experiments and Comparison

Comparing filtering results for a standard surface is helpful for highlighting the advantages or shortcomings of particular filtering algorithms. The comparison to be presented here will use both simulated and practical profiles.

**Fig. 1:**
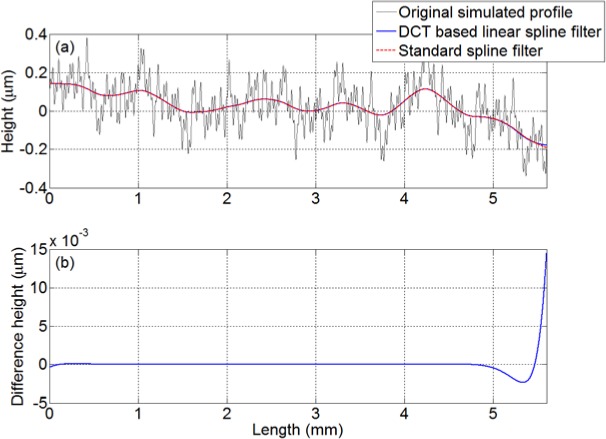
Simulated profile and mean lines: (a) DCT based linear spline filter and standard spline filter under cut-off wavelength 0.8 mm; (b) Difference of the mean line of the DCT based spline filter relative to the standard filter.

Figure 1 shows an experiment that assesses the linear filtering techniques. A simulated profile composed of 10 harmonic components with frequencies ranging from 0.1/*λ_c_* to 25/*λ_c_* is created, where the cut-off wavelength *λ_c_* equals 1600 points. The DCT based linear spline filter and standard spline filter were applied to this simulated profile. The filtering results and differences between the filtered profiles are shown in Figs. 1 (a) and (b) respectively. Figure 1 (b) clearly shows that the mean lines obtained by applying these two filters are almost identical, except at the ends of the surface. The identical results over most of the surface demonstrate the validity and feasibility of the new filtering algorithm. Also, the distortion occurring on both ends demonstrates the effect of the 1^st^ differential boundary condition. In Fig. 1 (a) the actual maximum deviation at the edges is 0.0145 µm, and the largest error is less than 2.02 % relative to the peak-to-peak value of the simulated surface. In fact, for a general measurement, this small error does not significantly impact the assessment of the profile as a whole.

**Fig. 2:**
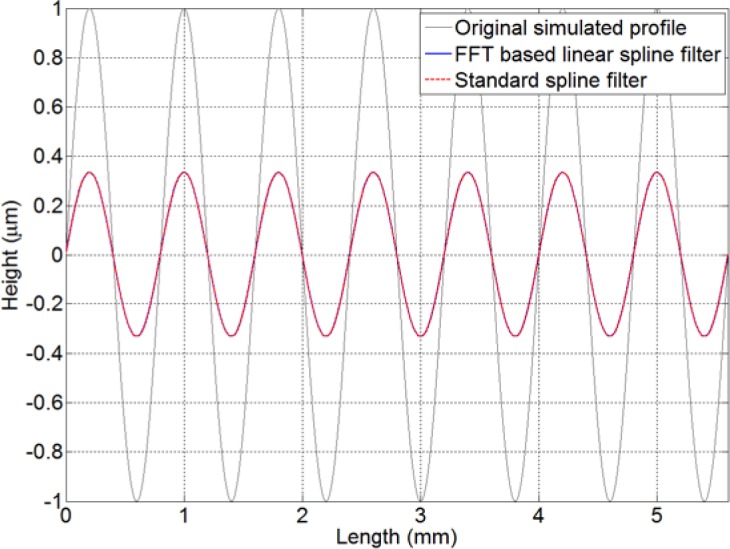
FFT based linear spline filter and standard spline filter for a periodic function using cut-off wavelength of 0.8 mm. The results from the FFT based algorithm and traditional matrix decomposition are indistinguishable.

In Fig. 2, a simulated periodic profile is used to validate the spline filtering algorithm based on the FFT. Both Eq. (12) and the standard spline filter with periodic boundary conditions are implemented. As expected, their filtered profiles are identical, even at the boundaries. This perfect result further demonstrates the accuracy and effectiveness of the new algorithm.

**Fig. 3:**
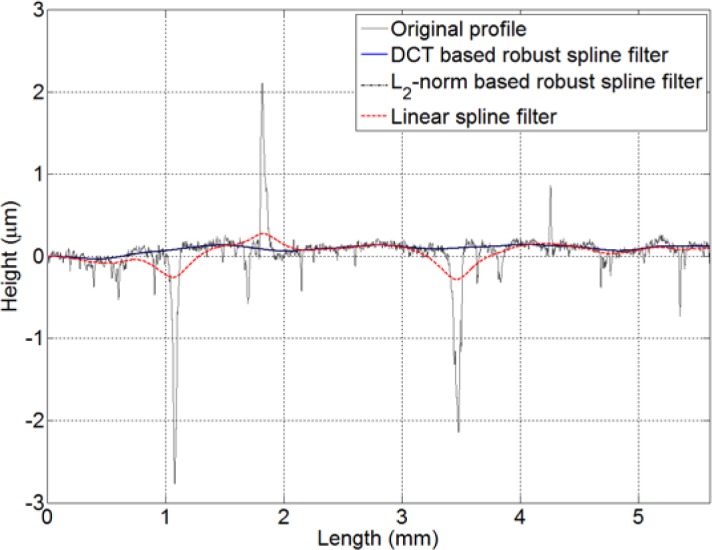
DCT based robust spline filter, L_2_-norm based robust spline filter and standard spline filter with a cut-off wavelength of 0.8 mm applied to data with outliers. In the graph, the filtered curves for DCT based robust spline filter and L_2_-norm based robust spline filter are indistinguishable in most parts.

Figure 3 shows a more complicated profile with a 5.6 mm length. It is found that there are many outliers such as scratches, deep valleys and sharp spikes distributed along this profile. The DCT based robust spline filter, the L_2_-norm based robust spline filter and the standard spline filter are applied to obtain the mean lines with a cut-off wavelength of 0.8 mm. It should be noted that the two robust filters can also easily handle missing data and dropouts if these points are assigned values that deviate far from the profile such that they appear as outliers. From the result in Fig. 3, it is evident that the robust algorithms are resistant to the outliers and able to acquire the satisfactory mean lines that follow the practical form very well.

**Fig. 4:**
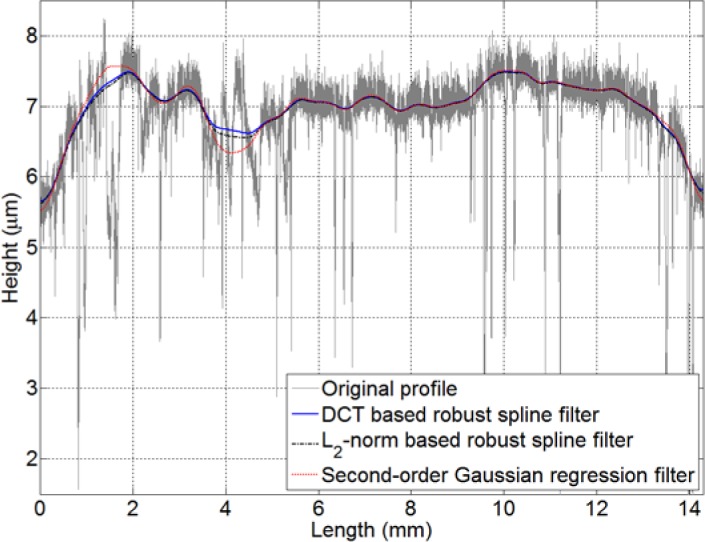
DCT based robust spline filter, L_2_-norm based robust spline filter and Gaussian regression filter with a cut-off wavelength of 0.8 mm applied to a practical profile.

In order to further demonstrate the efficacy of the newly proposed filter, an example consisting of a profile that may be encountered in practice is shown in Fig. 4. This practical engineering profile is 14.3 mm in length with sampling interval 0.125 µm. In this figure, the mean lines determined by the DCT based robust spline algorithm, the L_2_-norm based robust spline filter and the second-order Gaussian regression filter with a cut-off wavelength of 0.8 mm are presented concurrently. The robustness of the DCT based algorithm throughout the entire profile is verified again, but a larger difference between the robust spline filters and the Gaussian regression filter is exhibited in this example. These differences are caused by the dissimilar transmission characteristics of the filters and the second-order polynomial fitting principle in the Gaussian regression filter [17].

For this example, all of the filtering algorithms were implemented in Matlab and executed on an ordinary, commercially available desktop computer. For the test profile in Fig. 4, which contains 114,400 data points, the second-order Gaussian regression filter requires multiple hours to attain the robustness requirement. In comparison, the L_2_-norm based robust spline filter requires 1160 ms to finish the task, and the newly developed DCT robust algorithm takes only 720 ms to filter this profile. While the speed enhancements of the proposed method are only marginal for the profiles investigated here, it is possible that more dramatic improvements can be realized for larger or higher dimension arrays. Nevertheless, the primary benefit of this method is the relative simplicity of computing Eq. (15) and (16) without the requirement of any matrix operation and manipulation.

## 6. Conclusion

In this paper, several new algorithms for the spline filters are proposed based on the DCT and FFT. Both the linear and robust spline filters for periodic and non-periodic profiles are considered. In the resulting algorithms, the DCT and FFT which are utilized in the filtering process require only (*n*log(*n*)) computations, whereas the traditional algorithm based on Cholesky factorization requires *O*(*n*^3^). In addition to the potential scaling benefits, the DCT and FFT produce a more straightforward and simple to implement algorithm than the standard spline filter based on matrix decomposition. Meanwhile, although an approximation for the non-periodic boundary condition is utilized, the error caused by the end effects does not affect the surface evaluation significantly. For periodic profiles, the new and faster FFT based algorithm showed no differences when compared to the traditional spline filter.

In brief, these new algorithms for generating spline filters, based on the discrete cosine and discrete Fourier transforms, provide measureable improvements in computational speed and demonstrate excellent robustness to outliers and dropouts that are expected in real world data. Comparison of this new approach with traditional methods directly demonstrates the utility of these new techniques.
